# Antecedents and Contextual Factors Affecting Occupational Turnover among Registered Nurses in Public Hospitals in Hong Kong: A Qualitative Descriptive Study

**DOI:** 10.3390/ijerph17113834

**Published:** 2020-05-28

**Authors:** Maria S. Y. Hung, Stanley K. K. Lam

**Affiliations:** School of Nursing, Tung Wah College, Kowloon 999077, Hong Kong, China; stanleylam@twc.edu.hk

**Keywords:** occupational turnover, nurses, qualitative, interview

## Abstract

Global increases in both population size and ageing have led to a drastic expansion in the demand for healthcare services. The shortage of nursing workforce capacity continues, posing immense challenges for the global healthcare system. We aimed to identify the antecedents and contextual factors that contribute to the decisions of occupational turnover from the clinical duties of registered nurses in public hospitals in Hong Kong. A qualitative descriptive design was used in this study. A total of 18 registered nurses who had resigned from public hospitals in Hong Kong and changed their occupations were recruited via convenience and snowball sampling methods. Data were collected through individual, semi-structured, and face-to-face interviews and were analyzed according to the content analysis approach. The antecedents and contextual factors that contributed to the registered nurses’ decisions regarding occupational turnover were identified from the collected data. These factors were classified into three overarching categories: (1) job dissatisfaction due to a tense work environment, (2) low motivation due to limited career opportunities, and (3) inadequate communication due to ineffective leadership. The identification of these antecedents and contextual factors could help healthcare service providers to develop strategies to enhance nurses’ commitment and engagement in their positions and eventually improve their retention. Based on these factors, healthcare sector policy makers could consider incorporating appropriate strategies into healthcare system policy.

## 1. Introduction

Global increases in both population size and ageing have recently led to a drastic expansion in demand for healthcare services. Increased expectations regarding the standards and quality of provided healthcare services and the greater complexity of healthcare tasks have placed further demands on healthcare professionals. However, various projections regarding the healthcare workforce have suggested that the growth in the service has not been enough to meet the increased needs of the global population [1]. These reported projections have consistently highlighted that, of various healthcare shortages, the lack of professional nurses in healthcare systems worldwide will be the most severe [2]. 

Over time, shortages in nursing workforce capacity have been a continuous and even a worsening issue that has been studied frequently in the literature. The inadequate staffing of nurses in clinical settings may cause various problems in the quality of care and provision of services. For instance, the insufficient presence of nurses in a clinical setting may lead to a significant increase in mortality among hospitalized patients [3]. Another study reported a significantly higher surgical wound infection rate among post-operative patients in settings with higher nurse to patient ratios [4]. A shortage of nurses places an intense workload on nurses working on the frontline, which may negatively affect their job satisfaction and induce occupational stress, burnout, and psychological distress [5]. 

Reports from developed regions worldwide indicate alarmingly high rates of turnover among nurses [2,6,7]. The turnover of nurses may affect the standard of care, especially in highly specialized clinical settings such as intensive care units and accident and emergency departments [6]. The consequences of nurse turnover might have unavoidable negative impacts on economic costs, nursing care outcomes, and patient outcomes as well [7]. This high turnover rate of nurses may hinder mentoring relationships among nurses, affecting the latter’s professional development [8]. In addition to the turnover of experienced nurses, reports have indicated that many fewer junior nurses are choosing careers in clinical settings [9]. One report revealed that more than 16% of nurses leave clinical settings within five years of graduation in the United States [10]. Similarly, local healthcare services in Hong Kong are facing challenges with staff turnover and shortages for years, posing a chronic problem [11,12]. The Hospital Authority of Hong Kong, which manages all 43 public hospitals and institutions in Hong Kong, is highly concerned about the enormous workload managed by the frontline nurses; a total of 1400 nurses resigned from their hospital in 2018 [13]. This issue has also aroused the attention of the local government. Each nurse in the local public hospital was reported to care for about 24 patients in a night shift duty, which is far beyond the international standard of a nurse to patient ratio [14]. In a local study investigating the reasons for nurses’ organizational turnover from public hospitals to private hospitals, the findings showed that inadequate staffing taking on a demanding workload and unsatisfactory relationships with colleagues and supervisors were the main reasons [11]. Hospitals are also experiencing increasing service demands due to an ageing population with a shortage of nurses, which may further deteriorate the quality of health service delivery in public hospitals for the local citizens [12]. In summary, this evidence suggests that the poor retention of nurses is both a global and local crisis that warrants further attention. 

Few studies have investigated the factors contributing to nurses’ decisions to leave their clinical duties [9,10,11]. Although several studies emphasized the issue of turnover among nurses, most investigations merely described the prevalence and likelihood of turnover [15,16]. Therefore, the workplace constituents and context that influence nurses’ decisions remain poorly understood. This study, which was conducted to fill the existing knowledge gap, aimed to identify the antecedents and contextual factors that have contributed to the decisions of occupational turnover from their clinical duties among registered nurses in public hospitals in Hong Kong.

## 2. Methods

### 2.1. Design

A qualitative descriptive approach was used in this study. The purpose of qualitative research is to achieve a thorough and better understanding of a phenomenon [17]. Qualitative descriptive studies are commonly used in and suitable for healthcare and nursing studies for describing the nature of poorly defined phenomena through the investigation of participants’ perceptions and experiences. Researchers usually proceed from a naturalistic point of view and investigate the phenomenon in a natural state [18]. According to Graneheim and Lundman, the concepts of the content analysis in a qualitative descriptive nursing study include the manifest and latent content, unit of analysis, meaning unit, condensing, abstracting, content area, code, category, and theme [19]. The purpose of conducting qualitative content analysis is to orderly convert an enormous amount of transcribed interview texts into a vastly systematic and concise summary of meaningful findings [20]. Content analysis is a flexible reflective process that recognizes and condenses meaning units, creating codes and categories with constant comparisons to the initial data to produce a comprehensive picture and establish patterns in the findings [21].

### 2.2. Participants

In the present study, individuals who had resigned from public hospitals in Hong Kong and changed their occupations were recruited. Convenience and snowball sampling were used to recruit participants from researchers’ personal networks and then to invite the early participants to recommend other study participants [20]. Snowball sampling is a nonprobability method that involves members of the population who are difficult to access [18]. We first recognized a group of people with the target attributes, and after data collection, they were encouraged to refer similar cases for the study. The following inclusion criteria were applied to the participants: (1) previous employment as a registered nurse in Hong Kong, (2) resignation from the position of a registered nurse, (3) change in career from nursing to another occupation, and (4) willingness to share experiences regarding resignation and occupational turnover decisions. Verbal agreement from potential participants was initially obtained via face-to-face or telephone contact, followed by emails sent to those who verbally agreed. An information sheet and a consent form were attached to each email. Then, the locations and times of subsequent interviews were confirmed with the participants by email or telephone. Individuals were excluded if they had continued to work as a nurse in a clinical setting or hospital after resignation. A total of 18 nurse participants, including 17 registered nurses and 1 advanced practice nurse who had worked at different public hospitals in Hong Kong, were recruited. The demographic information on these participants is summarized in Table 1.

### 2.3. Data Collection

Data were collected through individual, semi-structured, face-to-face interviews. Individual in-depth interviews are one of the powerful methods for establishing an understanding of participants’ views. The interviewer allows the participants to express valuable information regarding personal experience in a freed, natural, and flexible way [22]. On the interview day, the participants were provided with a hard copy of the information sheet and consent form. Signed consent forms were collected from the participants after the researchers had explained the purpose of the study. Before the data collection interviews, the participants were also asked to provide demographic data, including gender, age, last rank in hospital, marital status, and the years of nursing experience when the previous hospital position was left. The interviews were conducted in Cantonese and audio-recorded with the permission and consent of the participants. 

The individual face-to-face interviews for data collection were conducted from January to April 2017 by two trained interviewers who were nursing professionals. An interview guide was used to guide the direction of each interview, which helped the participants to provide information deemed relevant to the research and to provide an understanding of the phenomenon of interest [23]. By using a simple interview guide, participants were asked to relate their experiences and feelings freely regarding their resignation decisions from clinical settings. It contained open-ended questions and non-leading questions intended to encourage participants to express their perceptions and opinions based on their personal experience of occupational turnover, e.g., “Please describe your nursing career development in the hospital before you left”, “What are the reasons/issues that made you decide to resign?”, and “Please describe your feelings and experience when you decided to leave your hospital clinical nursing job”. The interview guide emphasized an offering of flexibility to the participants rather than the rigidity of a checklist. 

During the interviews, the interviewers also used probing questions to encourage the participants to clarify unclear and ambiguous ideas that arose during conversations and to supplement essential concepts from their narrative. The following are examples of probing questions used in the course of the interviews: “Can you further explain the ideas you just mentioned?”, “Please tell me more about the situation you just mentioned”, and “How do you make sense of the issue you just mentioned?” Throughout the interviews, the participants’ emotional status was carefully observed. Field notes were written to document participants’ vocal intonations, physical expressions, and the researcher’s impressions of the ideas and concepts provided by the participants. These notes were then incorporated into the verbatim transcripts. Data saturation was targeted and achieved to ensure the comprehensive capture of relevant information [24]. After conducting the 18 interviews, data saturation occurred due to no new data being found. The duration of these interviews ranged from 40 to 92 min.

### 2.4. Data Analyses

We focused on using content analysis to gain a comprehensive and in-depth understanding of the context that surrounded these nurses and the influences of their decisions regarding occupational turnover. Before data analysis, the participants’ audio recordings were transcribed, and the first two transcribed interviews were reviewed regarding interview skills and text accuracy and necessary adjustments were made, such as avoid the use of leading/directive interview questions or probing questions and the avoidance of abrupt interruption to the participants’ conversation. Data analysis and data collection were conducted simultaneously, and these two processes were iterated and indivisible [25]. We individually scrutinized the transcripts line-by-line to capture relevant ideas, which were considered as meaningful units and codes. Ideas that shared similar characteristics were further collated and grouped into categories according to their properties. Through the condensation process, the texts were shortened, but the essential meaning was retained. Categories found to exhibit similar properties and dimensions were linked to form categories and subcategories. These clusters of concepts were considered more comprehensive and explicit when subcategories were used to represent and supplement the various properties of the categories.

### 2.5. Ethical Considerations

Ethical approval for this study was obtained from the School Research Committee of Tung Wah College prior to data collection. The entire research process adhered to the ethical principles for medical research delineated in the Declaration of Helsinki [26], which guarantees the principles of respect for persons, beneficence, and justice. The participants’ privacy and confidentiality were maintained and protected. All participant data were anonymized, and all possible identifiers were removed. All study information was sealed in a drawer with a padlock, and digital information (e.g., audio recordings and transcripts) was stored in a passcode-protected desktop computer.

## 3. Results

The antecedents and contextual factors that contributed to the nurses’ decisions regarding occupational turnover were identified from the data and classified into three overarching categories: (1) job dissatisfaction due to a tense work environment, (2) low motivation due to limited career opportunities, and (3) inadequate communication due to ineffective leadership. The codes, the subcategories, and the categories are summarized in Table 2.

### 3.1. Job Dissatisfaction Due to a Tense Work Environment

In this category, the participants expressed a job dissatisfaction at work, which was attributed to the tense and stressful work environment in the clinical settings of public health facilities. Almost all participants (16 out of 18) highlighted the intense healthcare delivery workload as a primary reason for their desire to leave their jobs as nurses and change to another occupation. One participant highlighted the problem of understaffing as follows:

*“In Hong Kong, the nurse to patient ratio has deviated so much from the international standard. We have to ensure both the efficiency and quality of care at the same time, so the tension and pressure are shifted to our shoulders. We all face a similar situation regardless of the specialty in which we work.”*

(Participant B)

Another participant stated that the workload associated with the position of a registered nurse was too intense, leading her to experience stress and frustration at work that affected her health and well-being. She said the following:

*“Providing healthcare delivery with such a massive workload does not merely exhaust me physically, but has also negatively affected my emotions. I consider it torture to go to work every day. I could not find any enjoyment or satisfaction in what I was doing. It was the main reason why I chose to leave this industry, and I will never go back.”*

(Participant F)

#### 3.1.1. Excessive and Additional Workload

In addition to the workload faced by registered nurses in the provision of healthcare services, the majority of the participants (14 out of 18) mentioned that the nature and scope of practice of a registered nurse are ambiguous, leading to an additional workload on top of their already tense routine practice, further aggravating their burdens. For example, one participant said the following:

*“My hospital frequently emphasized that we nurses had to treat our patients like our guests, and we should offer them warm hospitality and suggest that they make themselves at home. We were asked to handle the relatives’ requests and complaints. Sometimes those requests were nonsense; perhaps they thought that they were the customers, and we were the waitresses. Time was spent and wasted in this manner. It is ridiculous.”*

(Participant A)

Seven participants explained that the duties of training new staff and mentoring junior staff were another source of additional workload. The participants found it challenging to handle these duties, as some thought that they also held a junior status and could barely handle their own duties. One nurse shared her experience as follows:

*“I was assigned as a mentor of a junior nurse who had just been allocated to our department. I was responsible for sharing my experiences and knowledge with her. But because she was a new staff member, I had to frequently observe her practices and skills to avoid mistakes or accidents. Otherwise, I would have been blamed for not taking care of her, and the fault would be on me, although I was not the one who did wrong. It was so stressful. I considered myself a junior at the time, and guiding a more junior staff member placed a psychological burden on me on top of the demanding workload. I did not think I was the right person to be a mentor.”*

(Participant E)

#### 3.1.2. Strained Relationships with Colleagues

Besides the additional workload, ten participants highlighted that strained relationships between colleagues led to tension in the workplace. According to these participants, unpleasant colleague relationships could adversely affect their satisfaction at work, thus creating an antecedent for occupational turnover. For example, this participant described her emotions when facing hostile colleagues in the workplace:

*“I decided to resign and leave this occupation because I did not enjoy working in such an environment. The distrustful relationships among colleagues were hard to address, and every day I had to prepare to be blamed or blame others. I felt so insecure, and I did not gain any sense of accomplishment by performing this kind of small coterie.”*

(Participant Q)

Another participant further attributed her decision regarding occupational turnover to strained relationships between colleagues, highlighting that the roots of these difficulties lay in the culture of blame and horizontal violence in the nursing profession:

*“Whenever any issues occurred in the workplace, my colleagues always pinpointed people, in a hostile manner. One could not survive if they refused to get along with the existing groups. It is the culture of nursing, and it would appear in other settings. I felt frustration and despair, as my efforts and hard work were useless. It is the reason why I decided to resign and change to another career.”*

(Participant P)

### 3.2. Low Motivation Due to Limited Career Opportunities

This category outlines the antecedents to the nurses’ low motivation and their decisions regarding occupational turnover concerning limited career opportunities. Thirteen participants valued the importance of career opportunities. They stated that the limited scope for self-development and advancement had negatively affected their motivation to continue in the field of nursing. For example, one participant described the effect of limited career opportunities in the workplace on their decision to leave their nursing career as follows:

*“When I resigned, I thought that I was young, and I would like to try other career paths, instead of being a nurse for good. I did not want to spend my entire life in hospitals, as it was not worth it. I knew I would not be promoted no matter how hard I worked because I realised that there was a long queue in front of me. More senior colleagues would be promoted instead of me. I would rather go for positions in which more effort would mean that I would receive more gain.”*

(Participant Q)

#### 3.2.1. Scanty Professional Development

Several participants (8 out of 18) stated that their previous workplaces were unable to accommodate their needs for professional development in their roles as registered nurses and that this insufficiency encouraged them to leave clinical nursing and change to another career. According to the participants, this situation was primarily due to the unavailability of sufficient resources to promote and support nurses’ training and development. One participant described her perception and experience of the inadequate resources available for professional development as follows:

*“I had worked in the same department for a certain period, but I did not witness any actual self-improvement after all these years of working. I was only asked to complete the tasks in front of me without thinking about my future prospects. No training and development were provided to me during my working years. Perhaps no one cared about my future, and I was only considered a worker instead of an asset.”*

(Participant C)

Another participant echoed this sentiment:

*“At that time, I was not able to see my future. No training, no education, only work. I only knew that if I decided to stay, I would end up trapped there without any good prospects. I would rather find an alternative and try something else. I believed there were better options for me than nursing.”*

(Participant E)

#### 3.2.2. Poor Career Advancement Opportunities

The limited opportunity for promotion in the workplace was identified as another contextual factor contributing to the participants’ decisions regarding occupational turnover. Nearly all (17 out of 18) of the participants agreed that promotion was a potent and robust motivator that led them to strive for excellence and remain in their positions. However, seven participants revealed that the likelihood of promotion was quite low, despite their diligence and loyalty. A participant who had worked as a nurse for 11 years before resigning and changing her occupation shared her experience as follows:

*“I worked very hard from the first day I was in this profession. It was not entirely because I wanted a promotion, but I certainly welcomed an opportunity because I thought it was a type of reward and recognition for my diligence. However, the manager told me that the vacancies for promotion were limited and that there would be no new vacancies in the short term. I was very disappointed and frustrated. I lost my incentive to continue my career. I then decided to help my father’s business instead of being a nurse.”*

(Participant E)

Another participant also shared the experience of poor career advancement opportunities for nurses in the workplace. He said:

*“Honestly, I applied for a promotion to “Advanced Practice Nurse” four or five times. And of course, none of those times was successful. I was rejected during the interview stage. My motivation was depleted, and I decided to leave this occupation.”*

(Participant K)

### 3.3. Inadequate Communication Due to Ineffective Leadership

The third category portrayed an association between the occupational turnover of nurses and the leadership style in workplaces. Fourteen participants stated that an ineffective leadership style could create a breakdown of communication between nurses and management. This prevented nurses from expressing their concerns and seeking assistance when needed. Nine participants also sensed unfairness arising from inadequate communication regarding decisions related to staff management and treatment. One participant highlighted that she resigned from a nursing position due to receiving unfair treatment:

*“Being treated unfairly was not a sporadic issue for me. Actually, I had encountered similar situations before in other work settings. The manager assigned my duties and responsibilities according to her personal decisions without seeking my opinions. I eventually decided to leave this place and the profession as I did not want to be treated unfairly anymore.”*

(Participant P)

#### 3.3.1. Inattentive Administration and Management

Nine participants described the administration of their workplaces as passive and rigid. In such environments, the nursing staff found it difficult to express their views and receive support from management. One participant reported sensing that her opinions and suggestions for improving care and staff arrangements were continuously ignored:

*“My ideas and suggestions were suppressed continuously by the managers of my department. Perhaps I was considered a troublemaker. Or perhaps it was difficult for the management to face and address the suggestions I provided. I was not comfortable being a follower without my own decisions, and that was why I left.”*

(Participant Q)

Another participant complained that the department and hospital where she worked as a nurse were inattentive to her contributions and commitment. She said the following:

*“I expected that my department or hospital would appreciate my efforts. However, this was merely wishful thinking. I was not as important as I thought I was. I did not feel that my department or the hospital treasured me as an effective staff member. I realised that what I had done was so meaningless. My intention to leave the profession was ignited at that moment.”*

(Participant M)

#### 3.3.2. Unfair Treatment and Arrangements

Seven of the participants further commented that unfair treatment and arrangements implemented by workplace management had driven them to leave their nursing positions. This unfair treatment and arrangements concerned the assignment of workplace duties and roles. One participant explained the situation from her experience as follows:

*“I was pregnant at that time and was assigned fewer night shift duties. I was quite thankful for such an arrangement. However, when I completed my maternal leave, the manager of my department requested me to compensate for the night shifts I had been spared during my pregnancy. The manager did not even explain this arrangement to me, and I was so angry because it was unreasonable and unfair.”*

(Participant P)

## 4. Discussion

The literature mostly emphasizes the general turnover decisions and intentions of nurses, without determining whether the participants prefer to change their workplaces and settings (i.e., organizational turnover) or to change to another discipline or career (i.e., occupational turnover) [15]. Most of the literature focused on the thoughts and intentions of nurses who were employed before deciding to leave [15,16] and seldom captured their actual experience of changing jobs or provided a full description of relevant data. Information collected from nurses who remained in their positions might not sufficiently capture their real experiences surrounding decisions to leave the profession. Accordingly, an examination of the antecedent factors experienced by nurses who have already left the profession would better represent the in-depth and authentic experiences of those who have left the occupation. The contextual factors that adversely affect nurses’ willingness to remain in their positions must be eliminated. This approach will support healthcare workers’ commitment and engagement in their careers [27], which have been suggested, along with enhanced satisfaction and motivation, to significantly bolster the retention of nurses in healthcare settings and prevent a dearth of talent [28]. 

According to literature reviews of nurse turnover [7,29], the major determinants of nurse turnover included three significant aspects: organization factors, individual factors, and career advancement [29]. In line with this literature, similar findings were identified here. This study’s participants identified stress in the work environment as a significant contextual factor that adversely affected their job satisfaction. The participants highlighted two primary sources of stress: additional workload and tension between colleagues. These findings are consistent with the literature, wherein studies of both organizational and occupational turnover frequently identified a heavy workload as a major reason underlying a nurse’s decision to resign from public hospitals [11,12,13,30]. A local qualitative study using narrative analysis illuminated twelve registered nurses’ reasons for an organizational move from public to private hospitals, such as an inadequate workforce and a demanding workload, fairness of the remuneration policy, relationship with supervisors, and stressors [11]. However, constrained resources and increasing demands for healthcare services may create challenges for significantly reducing the workloads of nurses in clinical settings. Alternatives to the direct removal of workloads should be considered. For example, nurses might be more willing to perform their duties and remain in their positions despite heavy workloads if actual incentives and compensation (e.g., additional allowances and days off) were provided [31]. 

In addition to the physical workloads associated with healthcare delivery, the participants identified strained colleague relationships as a primary source of workplace tension and stress and a factor influencing their job satisfaction and intent to resign. These findings were echoed in the literature, suggesting that hostile relationships between colleagues in the workplace can negatively affect the retention of nurses [32]. Both the participants in this study and those in the literature frequently reported horizontal violence and workplace bullying within the nursing profession. These unhealthy practices must be addressed to prevent them from becoming part of the work culture of the nursing profession [32]. 

The study’s findings further revealed that inadequate career development opportunities contributed to nurses’ decisions regarding job resignation and career turnover. The participants considered career development to be an opportunity for further training and promotion. Similarly, the literature mentions promotion and advancement as a crucial milestone in the career development of nurses, who consider these events to be a recognition of and reward for their commitment [33]. Opportunities for professional development are essential for preparing a nurse for promotion. Therefore, strategies should be carefully devised to accommodate nurses’ desire for career development and thus maintain their motivation within and enthusiasm for the profession while improving retention. The importance of continuous education and training should be highlighted when evaluating opportunities for professional development. According to the literature, nurses are frequently required to spare their own time and money for continuous education and training and receive insufficient support from healthcare facilities [34]. Hospital administrations might consider providing more support to nurses, which would encourage their participation in further training and education. The nursing profession ranking could be reviewed to better differentiate the duties of staff members in the context of complicated and comprehensive healthcare delivery requirements. This structural review may lead to the creation of new positions, including additional vacancies for advancement, which would encourage morale among nurses and provide incentives for job commitment and engagement.

In addition to a stressful work environment and inadequate opportunities for development, the study’s participants identified ineffective leadership as an essential factor leading to their decisions regarding resignation and turnover. This is similar to the results of a local survey of 1271 registered nurses in 10 public hospitals that identified management and ward practice as the major factors in predicting nurses’ turnover intention [30]. The literature suggests that an ineffective management or leadership style (e.g., autocratic management) is associated with an increased turnover rate among nurses [30,35]. Some reports described the poor treatment of nurses and other instances of unfairness in healthcare settings, which highlighted the importance of proper leadership and management [29,30]. However, previous studies mostly identified this factor as a contributor to organizational turnover in public hospitals [10,11,30]. Few studies investigated the association between leadership style and occupational turnover [15,16,30]. Besides, governments and healthcare sectors must formulate timely and appropriate plans to address this severe nurse shortage crisis in public hospitals and their potential consequences [2,12,13]. Such policies could aim to establish an adequate supply of nurses for direct patient care via the recruitment and retention of professional nurses in the healthcare sectors. However, nurses are increasingly leaving the field of direct patient care despite the expansion of professional nurse training facilities intended to ensure an adequate supply of newly graduated nurses every year [2,12,13].

This study has several limitations, one of which is the small sample size in this qualitative study (18 participants from different hospitals), which may not be representative for drawing generalizable conclusions. However, as mentioned, the purpose of qualitative research is to achieve a thorough and better understanding of a phenomenon; caution in the identification of research samples can facilitate a more in-depth evaluation [18]. Another limitation is the timing of data collection. Some of the participants had been away from the nursing profession for prolonged periods. Therefore, their immediate situations may have differed from those of nurses who had recently resigned and changed their career paths. Additionally, the data were collected after the participants had resigned. In terms of their emotional reactions and perceptions of their experiences, this could lead to the risk of recall bias. Occupational turnover and organizational turnover were not fully differentiated in the data collected from the participants, possibly due to similarities in practice between nursing and other professions such as clinical teaching or healthcare-related project management. However, the findings may also have been influenced by the position, the duration of nursing experience of the participants, and the ongoing culture of the healthcare sector in Hong Kong. Furthermore, discussion and agreement among the interviewers and the co-researchers should be sought, to validate the sorted data and minimize subjective interpretation [19]. Despite these limitations, these findings help us to understand the decision to leave a career from the perspective of nurses who actually resigned.

## 5. Conclusions

The shortage of nurses is a significant challenge for healthcare systems worldwide and thus warrants attention. In this study, we identified several antecedents and contextual factors surrounding nurses’ decisions regarding occupational turnover: job dissatisfaction due to a tense work environment, low motivation due to limited career opportunities, and inadequate communication due to ineffective leadership. The identification of these possible antecedents and contextual factors could guide the development of strategies to enhance nurses’ commitment and engagement in their positions and eventually improve their retention. Service providers could use this information to devise strategies such as better professional training and development and more effective management and leadership skills to improve nurses’ satisfaction, motivation, and communication in the workplace. Based on the identified factors, healthcare sector policy makers could incorporate appropriate strategies into healthcare system policy. Further research might investigate the impact of nurse turnover on patients and health care sectors from their perspectives.

## Figures and Tables

**Table 1:** Demographic information of participants (*n* = 18).

Participant	Gender	Age (Years)	Marital Status	Job Position	Nursing Experience (Years)
A	M	31–35	Single	Registered Nurse	8
B	F	41–45	Married	Advanced Practice Nurse	18
C	F	36–40	Married	Registered Nurse	14
D	M	36–40	Single	Registered Nurse	3
E	F	31–35	Married	Registered Nurse	11
F	F	26–30	Married	Registered Nurse	4
G	F	41–45	Single	Registered Nurse	20
H	F	31–35	Married	Registered Nurse	8
I	F	26–30	Single	Registered Nurse	3
J	F	36–40	Married	Registered Nurse	6
K	M	36–40	Married	Registered Nurse	8
L	F	41–45	Married	Registered Nurse	16
M	F	26–30	Single	Registered Nurse	4
N	F	26–30	Married	Registered Nurse	5
O	F	Over 50	Married	Registered Nurse	14
P	F	31–35	Married	Registered Nurse	7
Q	F	41–45	Married	Registered Nurse	18
R	M	41–45	Single	Registered Nurse	21

**Table 2:** The categories, the subcategories, and the codes.

Categories	Subcategories	Codes
Job dissatisfaction due to a tense work environment	Excessive and additional workload	Problem of understaffing High nurse to patient ratio Training new staff and mentoring junior staff Work pressure on frontline staff Physical exhaustion Negative emotions, e.g., stress and frustration The distrustful relationship among colleagues Unpleasant colleague relationship Blaming culture and horizontal violence No job satisfaction nor enjoyment of work
	Strained relationships with colleagues	
Low motivation due to limited career opportunities	Scanty professional development	No further training or education chances Inadequate resources for professional development No sense of accomplishment Limited career advancement Dissatisfied with promotion culture Low promotion chance The unfair career advancement system Lost incentive to continue the nursing career Depleted motivation
	Poor career advancement opportunities	
Inadequate communication due to ineffective leadership	Inattentive administration and management	Inattentive to contributions and commitment Difficulty in expressing views and opinions Inadequate communication and support Ignoring staff opinions Decisions made without seeking staff’s opinion Unfair arrangement of duties and responsibilities No explanation for the arrangement
	Unfair treatment and arrangements	
